# Changes in pain knowledge, attitudes and beliefs of osteopathy students after completing a clinically focused pain education module

**DOI:** 10.1186/s12998-018-0212-0

**Published:** 2018-10-19

**Authors:** Kylie Fitzgerald, Michael Fleischmann, Brett Vaughan, Kevin de Waal, Sarah Slater, John Harbis

**Affiliations:** 1 College of Health & Biomedicine, Victoria University, Melbourne, Australia; 2 Department of Medical Education, Melbourne Medical School, University of Melbourne, Melbourne, Australia

**Keywords:** Manual therapy, Clinical education, Chronic pain, Acute pain, Measurement, Reliability estimation, Simulated learning, Assessment, Medical education

## Abstract

**Background:**

Chronic pain is a substantial burden on the Australian healthcare system with an estimated 19.2% of Australians experiencing chronic pain. Knowledge of the neurophysiology and multidimensional aspects of pain is imperative to ensure health professionals apply a biopsychosocial approach to pain. Questionnaires may be used to assess learner changes in neurophysiology knowledge and beliefs and attitudes towards pain after education interventions.

The aim of this study was to evaluate changes in pain neurophysiology knowledge, beliefs and attitudes following a 12 week clinically-focused pain module in year 3 osteopathy students as measured by the Neurophysiology of Pain (NPQ) Questionnaire and Health Care Providers Pain and Impairment Relationship scale (HC-PAIRS).

**Methods:**

A pre-post design was utilised. Learners completed a demographic information survey pre-module, and completed the NPQ & HC-PAIRS prior to undertaking, and after completing, a twelve week clinically-focused pain module.

**Results:**

Learners (*n* = 55) completed the NPQ & HC-PAIRS at both time points. The median NPQ score was significantly increased with a large effect size (*p* < 0.001, z = − 5.71, *r* = 0.78) following the completion of the module. In contrast, the HC-PAIRS total score was significantly increased after the completion of the module (*p* < 0.01, z = − 6.95, *r* = 0.91) suggesting an increase in negative pain attitudes and beliefs. Results indicate that a clinically-focused pain module can increase pain neurophysiology knowledge. However the HC-PAIRS results suggest an increase in negative pain attitudes and beliefs. The HC-PAIRS questionnaire was developed for use with chronic low back pain attitudes & beliefs in practitioners, rather than pre-clinical students. Students were provided with general principles of pain management, rather than condition specific pain management. This study is the first comparing pain neurophysiology knowledge and changes in attitudes and beliefs towards pain pre-post a clinically-focused pain module using the NPQ & HC-PAIRS.

**Conclusions:**

There was a significant improvement in NPQ score after the 12 week clinically-focused pain module. The HC-PAIRS result was paradoxical and may reflect issues with the module design or the measurement tool. The module duration is longer than that reported in the literature and demonstrates effectiveness in increasing pain neurophysiology knowledge.

**Electronic supplementary material:**

The online version of this article (10.1186/s12998-018-0212-0) contains supplementary material, which is available to authorized users.

## Background

Chronic pain is a substantial burden on the Australian healthcare system with an estimated 19.2% of Australians experiencing chronic pain [1]. The prevalence of chronic pain supports the need to develop an understanding of the multidimensional nature of pain so as to provide targeted care for the individual patient. Developing this understanding in health profession pre-registration training is important as it may assist in reducing the perception of pain as a structural and/or biomechanical issue [2], encourage guidelines adherence [3] and improve provision of information and advice to patients with respect to pain [4, 5].

The IASP has designated 2018 as the Global Year for Excellence in Pain Education [6]. This group suggests it is essential that health professionals undertake comprehensive pain education to ensure they are able to develop an appropriate biopsychosocial management approach tailored to the individual patient. Health profession students must develop thorough knowledge and understanding of these influences and how they may impact the individual to provide effective, ethical pain management [6, 7]. Evaluating acquisition and application of pain knowledge and evolution of attitudes and beliefs towards appropriate pain management is a critical component of assessing efficacy of pain curricula.

The systematic review by Ung et al. [8] identified multiple strategies to evaluate pain knowledge, attitudes and perceptions in medical and nursing students including a variety of questionnaires and clinical examination performance. This work suggests there is no one method by which pain knowledge, attitudes and perceptions can be evaluated.

Pain knowledge has been evaluated in multiple studies with health profession students using the Neurophysiology of Pain Questionnaire (NPQ) in either its 19-item or 13-item formats. The NPQ was developed to evaluate understanding of pain neurophysiology and the efficacy of short-term pain education programs [9]. Improvements in pain neurophysiology knowledge have been shown in physiotherapy students undertaking a short-term (80 min) neurophysiology pain course [10]. Whether the NPQ can accurately assess longer term acquisition of pain knowledge has not yet been investigated.

Multiple studies have evaluated pain beliefs and attitudes with a particular focus on low back pain [3, 11–13]. These studies have used multiple questionnaires to explore these attitudes and beliefs in different professions including physiotherapy, chiropractic and osteopathy. The questionnaires include the Health Care Providers’ Pain and Impairment Relationship Scale (HC-PAIRS) [14]. Attitudes to Back Pain Scale for musculoskeletal practitioners (ABS-mp) [15] and the Pain Attitudes and Beliefs Scale for Physiotherapists (PABS.PT) [12] among others.

The HC-PAIRS is the most widely used of these attitudes and beliefs questionnaires, and demonstrates acceptable internal consistency and retest reliability [11]. The questionnaire contains 15 items with respondents evaluating each item on 7 point Likert scale [14]. A number of studies evaluated attitude differences following pain knowledge and attitude educational interventions [2, 5, 16, 17], differences in attitudes between professions [3, 18–20] and differences between within-profession year levels [13, 18, 19] and countries [21–23]. These studies suggest that pain attitudes improve following education interventions, health profession students hold more positive beliefs about chronic low back pain than non-health profession students, and students in older year levels demonstrate more positive beliefs compared to younger students.

There is no research on the impact on health profession student pain neurophysiology knowledge, attitudes or beliefs with a longer-term and/or clinically-focused pain education module. The module developed in this study was developed using the IASP Curriculum Outline on Pain for Physical Therapy, and adapted for osteopathic practice. The adaptations involved removing elements of the recommended IASP curriculum that were not considered relevant for a pre-clinical osteopathy curriculum (e.g. specific types of exercise therapy, pharmacological or electro-physical agents).

### Objectives

1. Whether the 12 week clinically focused pain module improved year 3 osteopathic students pain neuroscience knowledge as measured by the NPQ;
2. Whether the 12 weeks clinically focused pain module improved year 3 osteopathic student’s pain attitudes and beliefs towards chronic low back pain as measured by the Health Care Providers Pain and Impairment Relationship scale (HC-PAIRS).

## Methods

This study was approved by the Victoria University Human Research Ethics Committee (HRE17–020).

### Participants

Learners enrolled in the clinically-focused pain module as part of the third year of the osteopathy course at Victoria University in 2017 were invited to participate in the study (*n* = 91).The module was commenced prior to the students undertaking their patient management role in the VU Osteopathy Clinic, a student-led teaching clinic in year 4 of the program. Completion of the questionnaires was not a requirement to satisfactorily complete the subject.

### Intervention

The 12 week clinically-focused pain module was developed to include pain neuroscience, assessment and measurement of pain, and evidence-based interventions for managing pain. The module learning outcomes were developed by three staff using the IASP Curriculum Outline on Pain for Physical Therapy: Objectives [7] as a curriculum development tool (Additional file 1). In accordance with the curriculum outline [9], the module was independent from other traditional modules such as orthopaedics, manual therapy or pharmacology and offered after students had completed courses in anatomy, physiology, biomechanics and most clinical skills courses (Table 1).

**Table 1:** – Module learning outcomes mapped to IASP Physical Therapy curriculum objectives

Module Learning Outcomes	IASP Curriculum Outline on Pain for Physical Therapy (2016) – Curriculum Objectives [9]
1. Relate the neuroanatomy and physiology to different types of pain presenting in clinical practice;	#1 Apply knowledge of basic science of pain to the assessment and management of people with pain.
2. Evaluate the impact of pain and consider influencing factors within the patient’s psychological and social context;	#2 Promote health and well-being through prevention of pain and disability. #4 Identify professional, system, patient, family and community barriers to effective pain assessment and management.
3. Conduct and interpret assessment of patients with specific types of pain, notably nociceptive/inflammatory pain, neuropathic pain and central sensitisation/amplification using clinical skills and outcomes measures;	#3 Assess and measure the biological and psychosocial factors that contribute to pain, physical dysfunction and disability using valid and reliable assessment tools. #7 Demonstrate an awareness of their scope of practice to evaluate and manage patients experiencing pain using evidenced-based practice strategies for clinical decision-making.
4. Critically review and apply the current research evidence for the use of manual therapy and its effects in pain treatment; and	#5 Develop an evidence-informed physical therapy management program in collaboration with the client/patient, directed at modifying pain, promoting tissue healing, improving function and reducing disability. #10 Practice in accordance with an ethical code that recognizes human rights, diversity, and the requirement to “do no harm.” #11 Reflect critically on effective ways to work with and improve care for people with pain. #12 Regularly update personal knowledge on pain and its management.
5. Plan osteopathic management aligning with patient’s pain presentation and include published tools for patient education and practical exercises.	#6 Implement management that includes patient education, active approaches such as functionally oriented behavioural movement re-education approaches and exercise (including pacing), and passive approaches such as manual therapy, and application of electro-physical agents as relevant. #8 When appropriate, refer patients in a timely manner for additional care to practitioners with expertise such as medical and surgical, behavioural and psychological, or pharmacological interventions.

The module included 12 h of online lectures accompanied by 8 × 1.5 h simulated learning activity workshops enabling learners to apply their knowledge to various acute and chronic musculoskeletal clinical presentations. These presentations addressed acute nociceptive (inflammatory and mechanical), sensitisation (central), and neuropathic pain scenarios. The final workshop enabled the learner to undertake a mock exam in the same format as the final exam.

The assessments included 4 online multiple choice quizzes – one formative, three summative (10% each = 30% final grade) at weeks 2, 3 and 4 to assess the neurophysiology content and provide feedback to the learner on their progress. The module content was tested by the final written examination (70% of final grade). The final exam was a classroom based assessment using a video of simulated patient acting out a common pain scenario with an osteopath asking and recording relevant presenting complaint and systems history questions. The scenario was a chronic pain presentation with central sensitisation as the dominant mechanism. The simulated patient also exhibited yellow flags including anxiety, catastrophizing, fear avoidance and compensable injury. Learners recorded a pain focused history from the video and used this information via question prompts to identify red & yellow flags and interpret completed, unscored outcome measures. The student was asked to identify and explain the dominant neurophysiology. Based on their previous answers and the history, students were then required to formulate an appropriate management plan.

### Data collection

Learners who volunteered to participate were asked to complete a short demographic information survey, the Neurophysiology of Pain Questionnaire (NPQ) and the Health Care Providers’ Pain and Impairment Relationship Scale (HC-PAIRS) [14] at the commencement of the semester (week 1). Learners then completed the twelve-week clinically focused pain module and prior the final workshop completed the NPQ and HC-PAIRS again (week 12).

### Data analysis

Data from the demographic questionnaire and pre- and post-intervention measures were entered into SPSS version 22 (IBM Corp, USA) for analysis. The NPQ has 19 questions and the user may choose from three options: correct, incorrect or undecided. The NPQ is scored out of 19 with 1 point awarded for each correct response. A score of 0 is attributed to both incorrect and undecided responses [9].

The HC-PAIRS has 15 questions, with a seven point scale of Likert responses of 1 (completely disagree) to 7 (completely agree) with a range of scores from 15 to 105. Items 1, 6 and 14 were inverted as per the authors instructions [14]. A lower HC-PAIRS total score suggest positive beliefs and attitudes that pain complaints do not justify impairments and disability [16].

Total scores for the pre- and post-module NPQ and HC-PAIRS scores were calculated. Descriptive statistics were generated for baseline demographic data and the pre- and post-intervention NPQ and HC-PAIRS items and total score. Non-parametric statistics were used to evaluate differences in the NPQ items and total score (Mann-Whitney U) and HC-PAIRS total score (Sign test) pre- and post-intervention (alpha set at *p* < 0.05) [2]. Spearman’s *rho* (ρ) was used to evaluate the relationship of age to the NPQ total score, and HC-PAIRS total score and difference score (time 2 minus time 1 total scores). Non-parametric effect sizes (r) were also calculated [24].

Data were exported to R [25] for the calculation of the reliability estimations (internal consistency) using the *psych* package [26]. Cronbach’s alpha (α) and McDonald’s omega hierarchical (ω_h_) and total (ω_t_) [27–29] were calculated. Cronbach’s α was generated for comparison with previous studies albeit the data in the present study do not meet the assumptions for the use of this statistic (tau-equivalency). Other authors have advocated that McDonald’s omega is a more appropriate reliability estimation [28, 30]. Cronbach’s α and McDonald’s ωt are interpreted in the same manner (acceptable value > 0.70) and McDonald’s omega hierarchical values over 0.50 suggest the calculation of a total score is acceptable [31].

## Results

Seventy (*n* = 70) learners completed the demographic questionnaire, NPQ and HC-PAIRS pre-intervention at the commencement of the week 1 lecture in semester 1, 2017. Matched pre-post NPQ and HC-PAIRS data were available for fifty-five (*n* = 55) of these participants. Demographic data is presented in Table 2. Data for each individual NPQ item is presented in Table 3.

**Table 2:** Demographic data for students participating in the study (matched data only)

Gender	
Male	26 (47.3%)
Female	29 (52.7%)
Age	
Mean (±SD) years	22 (±3.1)
Range	20–36 years
Previously undertaken a course or professional development activity related to pain and/or pain education	
Yes	2 (3.6%)
No	53 (96.4%)
Previously undertaken a course to become a health professional	
Yes	10 (18.2%)
No	45 (81.8%)
Personally experienced chronic persistent pain for more than 6 months	
No	32 (58.2%)
Previously experienced	14 (25.5%)
Currently experiencing	8 (14.5%)
Current & previous experience	1 (1.8%)

Note: percentages that do not add to 100% represent missing data

**Table 3:** Responses for the Neurophysiology of Pain (NPQ) questionnaire items (matched data only)

	Pre-intervention		Post-intervention	
NPQ item	Correct responses	Undecided responses	Correct responses	Undecided responses
1. Receptors on nerves work by opening ion channels in the wall of the nerve.	44 (80%)	10 (18.2%)	48 (88.9%)	3 (5.5%)
2. When part of your body is injured, special pain receptors convey the pain message to your brain.	1 (1.8%)	0 (0%)	3 (5.5%)	0 (0%)
3. Pain only occurs when you are injured or at risk of being injured.	37 (67.3%)	5 (9.1%)	40 (72.7%)	1 (1.8%)
4. Special nerves in your spinal cord convey “danger” messages to your brain.	42 (76.4%)	9 (16.4%)	46 (83.6%)	2 (3.6%)
5. Pain is not possible when there are no nerve messages coming from the painful body part.	26 (47.3%)	8 (14.5%)	36 (65.5%)	3 (5.5%)
6. Pain occurs whenever you are injured.	35 (63.6%)	7 (12.7%)	41 (74.5%)	3 (5.5%)
7. The brain sends messages down your spinal cord that can change the message going up your spinal cord.^	29 (52.7%)	14 (25.5%)	44 (80.0%)	5 (9.1%)
8. The brain decides when you will experience pain.*	34 (61.8%)	9 (16.4%)	48 (87.3%)	3 (5.5%)
9. Nerves adapt by increasing their resting level of excitement.	42 (76.4%)	9 (16.4%)	49 (89.1%)	4 (7.3%)
10. Chronic pain means that an injury hasn’t healed properly.	28 (50.9%)	7 (12.7%)	38 (69.1%)	4 (7.3%)
11. The body tells the brain when it is in pain.^	11 (20.0%)	7 (12.7%)	31 (56.4%)	4 (7.3%)
12. Nerves can adapt by producing more receptors.^	31 (56.4%)	14 (25.5%)	47 (85.5%)	14 (25.5%)
13. Worse injuries always result in worse pain.	39 (70.9%)	7 (12.7%)	46 (83.6%)	2 (3.6%)
14. Nerves adapt by making ion channels stay open longer.^	21 (38.2%)	31 (56.4%)	41 (74.5%)	8 (14.5%)
15. Descending neurons are always inhibitory.^	16 (29.1%)	38 (69.1%)	44 (80.0%)	8 (14.5%)
16. When you injure yourself, the environment that you are in will not affect the amount of pain you experience, as long as the injury is exactly the same.	44 (80.0%)	6 (10.9%)	51 (92.7%)	2 (3.6%)
17. It is possible to have pain and not know about it.	15 (27.3%)	5 (9.1%)	16 (29.1%)	4 (7.3%)
18. When you are injured, special receptors convey the danger message to your spinal cord.	40 (72.7%)	8 (14.5%)	44 (80.0%)	3 (5.5%)
19. All other things being equal, an identical finger injury will probably hurt the left little finger more than the right little finger in a violinist but not a piano player.	11 (20.0%)	26 (47.3%)	7 (20.0%)	15 (27.3%)
Total score (median & range)	10 (4–16)		14 (7–19)	

^*p* < 0.001, *p < 0.01

The median NPQ score increased from pre- (10/19 correct answers) to post-intervention (14/19 correct answers) with a large effect size (*p* < 0.001, z = − 5.71, *r* = 0.78). Significant increases in ‘correct’ responses were observed for items 7, 8, 11, 12, 14 and 15 (Table 3). The magnitude of the difference between the pre- and post-intervention NPQ total score was weakly related to age (ρ = 0.15), and no statistically significant differences were observed for gender, previous professional development in pain, undertaking training to be a health professional or personal experience with chronic pain (*p* > 0.05).

The HC-PAIRS descriptive statistics are presented in Table 4. The median HC-PAIRS total score significantly increased pre (46/105) to post (65/105) intervention with a large effect size (*p* < 0.001, z = − 6.79, *r* = 0.91). Two items of the HC-PAIRS items were significantly different pre-post with the median score reducing for both item 2 (*p* < 0.001, z = − 3.48, *r* = 0.47) and item 8 (*p* = 0.003, z = − 2.96, *r* = 0.40). All other items were not statistically significantly different. Age was weakly related to the magnitude of the difference between the pre- and post-intervention HCPAIRS total score (ρ = − 0.16), and no statistically significant differences were observed for gender, previous professional development in pain, undertaking training to be a health professional or personal experience with chronic pain (*p* > 0.05).

**Table 4:** Descriptive statistics for the HC-PAIRS at time 1 and time 2

Item	Pre-Intervention		Post- Intervention	
	Median	Range	Median	Range
1. Chronic back pain patients can still be expected to fulfil work and family responsibilities despite pain.^a^	3	1–6	3	1-7
2. An increase in pain is an indicator that a chronic back pain patient should stop what he is doing until the pain decreases.	6	2–7	5	1–7
3. Chronic back pain patients cannot go about normal life activities when they are in pain.	5	2–7	5	1–7
4. If their pain would go away, chronic back pain patients would be every bit as active as they used to be.	4	2–6	4	1–7
5. Chronic back pain patients should have the same benefits as the disabled because of their chronic pain problem.	4	1–6	4	1–6
6. Chronic back pain patients owe it to themselves and those around them to perform their usual activities even when their pain is bad.^a^	5	3–7	5	2-7
7. Most people expect too much of chronic back pain patients, given their pain.	5	2–7	4	1–7
8. Chronic back pain patients have to be careful not to do anything that might make their pain worse.	6	2–7	5	1–7
9. As long as they are in pain, chronic back pain patients will never be able to live as well as they did.	5	1–7	5	1–7
10. When their pain gets worse, chronic back pain patients find it very hard to concentrate on anything.	6	3–7	5	3–7
11. Chronic back pain patients have to accept that they are disabled persons due to their chronic pain.	3	1–6	2	1–5
12. There is no way that chronic back pain patients can return to doing the things that they used to do unless they first find a cure for their pain.	3	1–6	3	1–6
13. Chronic back pain patients find themselves frequently thinking about their pain and what it has done to their life.	5	2–7	5	2–7
14. Even though their pain is always there, chronic back pain patients often don’t notice it at all when they are keeping themselves busy.^a^	4	2–6	4	1-6
15. All of chronic back pain patients problems would be solved if their pain would go away	3	1–6	3	1–7
Total score	46	32–55	65	39–86

^a^rescored

Cronbach’s alpha for the HC-PAIRS both pre- (α=0.75, 95%CI 0.66–0.85) and post-intervention (α=0.83, 95%CI 0.76–0.89) was acceptable however the statistic suggested that the removal of items 4, 6 and 14 would improve the value to 0.76 pre-intervention, and removing item 4 post-intervention would improve this to 0.85. McDonald’s omega hierarchical was acceptable pre- (ω*t* = 0.79, ωh = 0.53) and post-intervention (ω*t* = 0.87, ωh = 0.68) suggesting a total score for the HC-PAIRS is valid.

## Discussion

This study sought to evaluate changes in year 3 osteopathy student’s pain neurophysiology knowledge, and their attitudes and beliefs towards pain following a 12-week clinically focused pain module. The module was developed using the curriculum objectives outlined by the IASP Curriculum Outline on Pain for Physical Therapy [7], adapted for osteopathic practice.

### Neurophysiology of pain questionnaire

Knowledge level was measured using the Neurophysiology of Pain Questionnaire (NPQ) and a significant increase was observed pre- and post-module with a large effect size. Previous studies evaluating short-term pain education programs with physiotherapy students demonstrate an improvement in their pain neurophysiology knowledge and understanding [17, 32, 33]. This improvement was consistent with the present study, with the median NPQ score rising from 53% (10/19) to 74% (14/19) of correct answers.

The increase in correct responses is consistent with Cox et al. [32]. Their study included 77 first year physical therapy students who demonstrated an increase in NPQ scores from 41% (8/19) to 84% (16/19) following a three-hour therapeutic neuroscience education lecture. There were no details provided about the curriculum delivered to enable comparison of content with this study.

Colleary et al. [17] demonstrated an increase of 45% (6/13) to 79% (10/13) after a 70-min didactic lecture with 72 first year physiotherapy students in the UK and Ireland using the Rasch-analysed NPQ [34]. The clinically-focused pain module in the present study included 12 h of theory and 15 h of simulated clinical learning activities. The learner cohort were commencing year 3 of their course and had completed two years of preclinical sciences, including anatomy, pathophysiology, and neurological system examinations, which may account for the higher commencing NPQ score. The timing of the module was informed by the IASP Curriculum Outline on Pain for Physical Therapy [7] which recommended students had completed courses in anatomy, physiology, biomechanics and most clinical skills courses prior to undertaking pain curricula.

A number of individual NPQ items showed significant increases in ‘correct’ responses for items 7, 8, 11, 12, 14 and 15 (Table 3). The improvement in these items may reflect the module focus on pain neurophysiology in weeks 1–4 of the program. This content was assessed via summative online MCQ quizzes (30% of total mark) at the end of each of these weeks.

Where minimal differences were observed, this could provide educators with an indication as to content that could either be included in a program of study, or strengthened in the clinically-focused pain module. Colleary et al. [17] and Cox et al. [32] did not report on score changes with individual items so it is unclear whether these studies observed a similar result, or whether the improvement was across all items.

### HC-pairs

The total HC-PAIRS score for this cohort increased significantly pre-post intervention indicating a decline in positive attitudes and beliefs towards patients with chronic low back pain [9]. This finding is in contrast to other student-based studies where reductions in the total score have been observed. For example, Latimer et al. [35] demonstrated a 6.6 point decrease after a 16-h pain workshop series on chronic low back pain. Further, the pre-intervention HC-PAIRS total score of 46 was lower than other studies with a score of 63.89 [36] and 57.2 [13] but consistent with other Australian health profession students (pooled HC-PAIRS of health students = 46.3 [3].

As described previously, the third year learners had already completed two years of pre-clinical sciences which may account for the initially positive attitude. The post intervention score showed regression of pain attitudes and beliefs compared with Domenech post-intervention [36]. A possible explanation may be due to the module content being spread across a 12 week period, compared to other research which has focused on short term (1–3 h) of education only, where the information may perhaps remain more accessible to the learner.

Other considerations may include the HC-PAIRS being designed for measurement of practitioners rather than students and thus may fail to capture attitudes or beliefs adequately in this cohort. The HC-PAIRS was also developed with a focus on chronic low back pain specifically, not pain in general. The module the learners undertook was not condition focused, but provided education on pain mechanisms and contributors in general that could be applied to a range of pain conditions. The specificity of the measure may have failed to capture general pain beliefs and attitudes in the cohort.

In contrast, two of the individual item HC-PAIRS scores were significantly different and showed a change to more positive attitudes and beliefs. Items 2 (An increase in pain is an indicator that a chronic back pain patient should stop what he is doing until the pain decreases) and item 8 (Chronic back pain patients have to be careful not to do anything that might make their pain worse) showed improvement. These two items relate to pain-driven behaviours suggesting that the clinically-focused pain module significantly improved the student’s attitudes towards the ability of a patient with low back pain to participate in activity. This positive attitude change is consistent with current guidelines with respect to continuing to move with low back pain and reflects content that was delivered in this topic. Future iterations of the clinically focused pain module may require an increase in content to support learners in changing beliefs and attitudes towards chronic back pain patients as this was not a specific learning outcome of the module.

### Demographic variables: NPQ & HC-PAIRS

The magnitude of attitude change was weakly negatively associated with age, and there were no significant differences for other demographic variables for the HC-PAIRS total score. No difference in HC-PAIRS for gender has also been reported by other authors [12, 18, 22] and multiple studies have demonstrated that personal experience with low back pain does not influence HC-PAIRS total scores [19, 21, 22]. Morris et al. [19] posit that it may be that training has a larger effect on attitudes and beliefs than personal experience.

Likewise, demographics largely do not appear to influence responses to either the NPQ. A weak relationship between age and NPQ score (ρ = 0.15) was observed. Adillon et al. [37] identified that male physiotherapy students demonstrated higher NPQ scores compared to females at the point of graduation from a physiotherapy program, however this difference was not observed in medical students. These authors suggested “…that men perceive better the biopsychosocial aspects of pain” (p.7) however such an assertion is not supported in the present study. Similar to Cox [32], no significant difference was observed between learners having personal experience of chronic pain (*n* = 9) and those who have not with regard to the total NPQ score both pre- and post-intervention.

### Limitations

A limitation of the present study appears to be the measurement properties of the HC-PAIRS. Domenech et al. [36] demonstrated a test-retest ICC of 0.50 with four weeks between administrations, whilst Rainville et al. [38] demonstrated a test-retest Pearson’s *r* of 0.64 with three months between administrations suggesting that responses to the questionnaire may not be stable over time. Both of the methods of reliability estimation demonstrated issues with respect to the individual items and the generation of a total score. These findings are in contrast to other authors who have demonstrated high Cronbach’s alpha values for the HC-PAIRS [36, 39, 40] but appears consistent with the original authors (α=0.78) [14] and others (α=0.74) [23]. Houben et al. [40] suggested the removal of items 10 and 13 in their work with a range of manual therapists to create a 13-item questionnaire, and Domenech et al. [36] suggested the removal of items 4 and 7 would improve the α score. The current study also identified item 4 as one that could be removed to improve the α score. Of note is the limited data on the measurement properties of the questionnaire particularly where authors have used it as a measure of their intervention. A number of studies have reported factor analyses [36, 40] however many studies that have used the measure have not reported rudimentary measurement data such as Cronbach’s α in their study population [2, 3, 18, 19, 22, 35]. Additional work appears to be required to evaluate the measurement properties in other health profession student and practitioner populations [11], and particularly the evaluation of these properties using item response theory approaches [36].

Other limitations of our study are the investigation of a single cohort at a single institution, and lack of a control group or alternative comparative intervention. As the participants completed the NPQ and HC-PAIRS during class time, the two most likely causes for drop out include; non-attendance or choosing not to fill out the survey.

Future research will evaluate pain knowledge levels in the same participants as part of a longitudinal study. There are also opportunities arising from the current work including measurement of stress, anxiety and/or fears in managing patients with persistent pain, and use of additional or alternative measures of the pain experience.

## Conclusion

The results of the current study suggest a 12 week clinically-focused pain module improves pain neurophysiology knowledge in year 3 osteopathy students however a similar positive change was not reflected in the attitudes to those with chronic low back pain. This may be related to the measurement properties of the questionnaire (i.e. low test-retest reliability) or the content of the module not focusing specifically on chronic low back pain as in other studies [35].

Further the module did not specifically address practitioner attitudes to pain and the results suggest that a module emphasising pain neurophysiology in a clinical context does not change these attitudes. The intervention developed and used in the present study could be readily implemented in other educational settings and appears to be effective in increasing knowledge. The addition of specific content related to change in pain attitudes may also be of benefit. Longer term follow-up is required to ascertain if these improvements are sustained, and potentially how these improvements may contribute to the management of patients with chronic pain.

## Additional file

Additional file 1: Weekly Plan for 12 week clinically focused pain module. (DOCX 18 kb)

## References

[1] Henderson JV, Harrison CM, Britt HC (2013). Prevalence, causes, severity, impact, and management of chronic pain in Australian general practice patients. Pain Med (Malden, Mass).

[2] Overmeer T, Boersma K, Main CJ (2009). Do physical therapists change their beliefs, attitudes, knowledge, skills and behaviour after a biopsychosocially orientated university course?. J Eval Clin Pract.

[3] Briggs A, Slater H, Smith A (2013). Low back pain-related beliefs and likely practice behaviours among final-year cross-discipline health students. Eur J Pain.

[4] Evans DW, Breen AC, Pincus T (2010). The effectiveness of a posted information package on the beliefs and behavior of musculoskeletal practitioners: the UK chiropractors, osteopaths, and musculoskeletal physiotherapists low back pain ManagemENT (COMPLeMENT) randomized trial. Spine.

[5] Moses J, Oliver G, Paul-Taylor G (2016). ‘Manage backs’ group intervention: applying a biopsychosocial explanation of low back pain at physiotherapy care pathway entry. European Health Psychologist.

[6] International Association for the Study of Pain (IASP). 2018 Global Year for Excellence in Pain Education 2018 [cited 2018 January 25th ]. Available from: https://www.iasp-pain.org/GlobalYear

[7] International Association for the Study of Pain (IASP). IASP Curriculum Outline on Pain for Physical Therapy 2018 [cited 2018 January 25th ]. Available from: https://www.iasp-pain.org/Education/CurriculumDetail.aspx?ItemNumber=2055

[8] Ung A, Salamonson Y, Hu W (2016). Assessing knowledge, perceptions and attitudes to pain management among medical and nursing students: a review of the literature. Br J Pain.

[9] Moseley L (2003). Unraveling the barriers to reconceptualization of the problem in chronic pain: the actual and perceived ability of patients and health professionals to understand the neurophysiology. J Pain.

[10] Mine K, Gilbert S, Tsuchiya J, Nakayama T. The Short-Term Effects of a Single Lecture on Undergraduate Physiotherapy Students’ Understanding Regarding Pain Neurophysiology: A Prospective Case Series. J Musculoskeletal Disord Treatment. 2017;3(41) doi: 10.23937/2572-3243.1510041

[11] Bishop A, Thomas E, Foster NE (2007). Health care practitioners’ attitudes and beliefs about low back pain: a systematic search and critical review of available measurement tools. Pain.

[12] Magalhães MO, Costa LO, Cabral C (2012). Attitudes and beliefs of Brazilian physical therapists about chronic low back pain: a cross-sectional study. Brazilian J Physical Ther.

[13] Quinn T, Ryan C, Jones D (2014). Physiotherapy Students’ attitudes towards the functional ability of patients with chronic low back pain. Pain Rehabilitation J Physiother Pain Assoc.

[14] Rainville J, Bagnall D, Phalen L (1995). Health care providers’ attitudes and beliefs about functional impairments and chronic back pain. Clin J Pain.

[15] Pincus T, Vogel S, Santos R (2006). The attitudes to back pain scale in musculoskeletal practitioners (ABS-mp): the development and testing of a new questionnaire. Clin J Pain.

[16] O’Sullivan K, O’Sullivan P, O'Sullivan L (2013). Back pain beliefs among physiotherapists are more positive after biopsychosocially orientated workshops. Physiother Pract Res.

[17] Colleary G, O’Sullivan K, Griffin D (2017). Effect of pain neurophysiology education on physiotherapy students’ understanding of chronic pain, clinical recommendations and attitudes towards people with chronic pain: a randomised controlled trial. Physiother.

[18] Ryan C, Murphy D, Clark M (2010). The effect of a physiotherapy education compared with a non-healthcare education on the attitudes and beliefs of students towards functioning in individuals with back pain: an observational, cross-sectional study. Physiotherapy.

[19] Morris H, Ryan C, Lauchlan D (2012). Do medical student attitudes towards patients with chronic low back pain improve during training? A cross-sectional study. BMC Med Edu.

[20] Ali N, Thomson D (2009). A comparison of the knowledge of chronic pain and its management between final year physiotherapy and medical students. Eur J Pain.

[21] Ferreira PH, Ferreira ML, Latimer J (2004). Attitudes and beliefs of Brazilian and Australian physiotherapy students towards chronic back pain: a cross-cultural comparison. Physiother Res Int.

[22] Alshami AM, Albahrani YA (2015). A comparison of the attitudes toward chronic low back pain in Saudi, Australian and Brazilian physical therapy students. J Taibah University Med Sci.

[23] Burnett A, Sze CC, Tam SM (2009). A cross-cultural study of the back pain beliefs of female undergraduate healthcare students. Clin J Pain.

[24] Yatani K. Mann-Whitney's U test 2016 [cited 2016 January 9]. Available from: http://yatani.jp/teaching/doku.php?id=hcistats:mannwhitney

[25] R Core Team. R: A language and environment for statistical computing Vienna, Austria: R Foundation for Statistical Computing; 2015; Version 3.2.2:[Available from: https://www.R-project.org/.

[26] Revelle W (2015). Psych; procedures for personality and psychological research. 1.5.4.

[27] Revelle W, Zinbarg RE (2009). Coefficients alpha, beta, omega, and the glb: comments on Sijtsma. Psychometrika.

[28] Zinbarg RE, Revelle W, Yovel I (2005). Cronbach’s α, Revelle’s β, and McDonald’s ω H: their relations with each other and two alternative conceptualizations of reliability. Psychometrika.

[29] Zinbarg RE, Yovel I, Revelle W (2006). Estimating generalizability to a latent variable common to all of a scale's indicators: a comparison of estimators for ωh. Appl Psychol Meas.

[30] Green SB, Yang Y (2009). Commentary on coefficient alpha: a cautionary tale. Psychometrika.

[31] Revelle W (1979). Hierarchical cluster analysis and the internal structure of tests. Multivar Behav Res.

[32] Cox T, Louw A, Puentedura EJ (2017). An abbreviated therapeutic neuroscience education session improves pain knowledge in first-year physical therapy students but does not change attitudes or beliefs. J Manual Manipulative Ther.

[33] Marques ES, Xarles T, Antunes TM (2016). Evaluation of physiologic pain knowledge by physiotherapy students. Revista Dor.

[34] Catley MJ, O'Connell NE, Moseley GL (2013). How good is the neurophysiology of pain questionnaire? A Rasch analysis of psychometric properties. J Pain.

[35] Latimer J, Maher C, Refshauge K (2004). The attitudes and beliefs of physiotherapy students to chronic back pain. Clin J Pain.

[36] Domenech J, Segura-Ortí E, Lisón JF (2013). Psychometric properties and factor structure of the Spanish version of the HC-PAIRS questionnaire. Eur Spine J.

[37] Adillón C, Lozano È, Salvat I (2015). Comparison of pain neurophysiology knowledge among health sciences students: a cross-sectional study. BMC Res Notes.

[38] Rainville J, Carlson N, Polatin P (2000). Exploration of physicians’ recommendations for activities in chronic low back pain. Spine.

[39] Moran RW, Rushworth WM, Mason J (2017). Investigation of four self-report instruments (FABT, TSK-HC, back-PAQ, HC-PAIRS) to measure healthcare practitioners’ attitudes and beliefs toward low back pain: reliability, convergent validity and survey of New Zealand osteopaths and manipulative physiotherapists. Musculoskelet Sci Pract.

[40] Houben RM, Vlaeyen JW, Peters M (2004). Health care providers’ attitudes and beliefs towards common low back pain: factor structure and psychometric properties of the HC-PAIRS. Clin J Pain.

