# Supplementary material for: Changes in pain knowledge, attitudes and beliefs of osteopathy students after completing a clinically focused pain education module
Source: Chiropr Man Therap. 2018 Oct 19;26:42. doi: 10.1186/s12998-018-0212-0 (PMC6194600; doi:10.1186/s12998-018-0212-0)
Supplement: Supplementary file 1 — Weekly Plan for 12 week clinically focused pain module. (DOCX 18 kb) [file 12998_2018_212_MOESM1_ESM.docx]

**Additional file 1 Weekly Plan for 12 week clinically focused pain module**

|  | **Weekly topics** | **LOs met** | **Workshops**  **(2 Hours x 9 sessions)** | **LOs met** | **Assessment** |  |
| --- | --- | --- | --- | --- | --- | --- |
| 1 | What is pain?  Nociceptive/Inflammatory pain: Neuroanatomy & physiology (2 hours) | 1 | NONE |  |  |  |
| 2 | Central Amplification/ Sensitization: Neuroanatomy & physiology (1 hour) | 1 | NONE |  | Quiz 1  formative |  |
| 3 | Peripheral Sensitization: Neuroanatomy & physiology (1 hour) | 1 | **Workshop#1**  Simulated patient introduction | 1,2 | Quiz 2 (10%)  Online MCQ |  |
| 4 | Neuropathic Pain (1 hr)  Neuroanatomy & physiology | 1 | **Workshop#2**  Taking a pain focused history (simulation) | 1, 3 | Quiz 3 (10%) Online MCQ |  |
| 5 | Nociceptive/Inflammatory pain (2 hours)   - Brief recap of definition - Yellow flags/psychosocial indicators - Impact on patient - Physical examination of patient & interpretation - Outcome measures - Evidenced based management including advice, first aid, hands on techniques, basic exercises | 2,3,4, 5 | **Workshop #3**  Review: sensory examination  Taking patient history and sensory examination scenario (simulation) | 1, 2, 3 | Quiz 4 (10%)  Online MCQ |  |
| 6 |  | 2,3,4, 5 | **Workshop #4**  Acute nociceptive /Inflammatory pain scenario 1 – history and examination, management (simulation) | 4,5 |  |  |
| 7 | Central Amplification (2 hours)   - Brief recap of definition - Yellow flags/psychosocial indicators - Impact on patient - Physical examination of patient & interpretation - Outcome measures - Evidenced based management including advice, first aid, hands on techniques, basic exercises | 2,3,4, 5 | **Workshop #5**  Acute nociceptive /mechanical pain scenario 2 – history and examination, management (simulation) | 1, 2, 3 |  |  |
| 8 |  | 2,3,4, 5 | **Workshop #6**  Chronic pain: central sensitization/amplification pain scenario pain scenario 3 – history and examination, management (simulation) | 4,5 |  |  |
| 9 | No lecture | 2,3,4, 5 | **Workshop #7**  Chronic pain: central sensitization/amplification pain scenario pain scenario 4 – history and examination, management (simulation) | 1, 2, 3, 4, 5 |  |  |
| 10 | Neuropathic Pain (2 hours)   - Brief recap of definition - Yellow flags/psychosocial indicators - Impact on patient - Physical examination of patient & interpretation - Outcome measures - Evidenced based management including advice, first aid, hands on techniques, basic exercises | 2,3,4, 5 | **Workshop #8**  Neuropathic pain: pain scenario pain scenario 5 – history and examination, management (simulation) | 1,2,3,4,5 |  |  |
| 11 | No lectures |  | **Workshop #9**  Deliberate Exam practice: students view simulated patient video, and answer same questions as exam | 1,2,3,4,5 |  |  |
| **Final Exam (70%)** | | | | | | |
